# Postoperative perfusion volume on territorial arterial spin labeling provides an exploratory risk signal for cerebrovascular autoregulation failure after moyamoya revascularization: a prospective physiological study

**DOI:** 10.3389/fphys.2026.1860201

**Published:** 2026-07-29

**Authors:** Zening Yang, Zhizhao Ma, Baogen Pan, Lijie Liu, Yeju He, Yankai Wu, Shiyuan Zhang

**Affiliations:** 1 Hebei Medical University, Shijiazhuang, China; 2 Department of Neurosurgery, Hebei General Hospital, Shijiazhuang, China; 3 Department of Neurosurgery, The Second Hospital of Hebei Medical University, Shijiazhuang, China; 4 Department of Neurology, The Eighth People’s Hospital of Hebei Province, Shijiazhuang, China; 5 Department of Radiology, The Second Hospital of Hebei Medical University, Shijiazhuang, China

**Keywords:** moyamoya disease, cerebral hyperperfusion syndrome, arterial spin labeling, revascularization, risk stratification

## Abstract

**Background:**

Cerebral hyperperfusion syndrome (CHS) after revascularization for moyamoya disease (MMD) represents a failure of cerebrovascular autoregulation—a system-level vascular physiological process. Non-invasive tools to quantify autoregulatory reserve and support early risk stratification remain limited. This study aimed to evaluate whether postoperative perfusion volume measured by territorial arterial spin labeling (tASL) provides an exploratory risk signal for cerebrovascular autoregulation failure after MMD revascularization and to identify candidate physiological factors associated with this complication.

**Methods:**

This prospective cohort study enrolled 116 adult MMD patients who underwent combined revascularization (suprasylvian approach, n=60; infrasylvian approach, n=56). All patients underwent 3D pseudo-continuous arterial spin labeling (3D-pCASL) preoperatively and postoperatively, and tASL on postoperative day 1. Postoperative perfusion volume and regional cerebral blood flow (CBF) were quantified. Firth’s penalized multivariable logistic regression was used to evaluate candidate factors associated with autoregulation failure (CHS) while avoiding statistical overfitting due to rare events (13 CHS cases). Receiver operating characteristic analysis determined exploratory threshold values. CHS was diagnosed contemporaneously during hospitalization by integrating new neurological symptoms, blood-pressure/hemodynamic context, and ASL evidence of focal postoperative hyperperfusion.

**Results:**

In the Firth penalized multivariable model, hypertension and Stage IV angiographic disease were associated with postoperative CHS, while Stage V showed a positive but statistically imprecise estimate. Age and tASL-derived postoperative perfusion volume showed exploratory ROC signals, with cutoffs of 61 years and 104.45 cm³, respectively. The suprasylvian approach showed a numerically higher early CHS rate than the infrasylvian approach (16.7% vs. 5.4%; Fisher exact P = 0.077), while showing higher regional CBF at postoperative day 30 and lower postoperative mRS scores at POD30. The low overall CHS incidence (13/116, 11.2%) should be interpreted in the context of standardized inpatient monitoring and active postoperative blood-pressure management.

**Conclusion:**

Quantitative tASL perfusion volume provides a non-invasive, exploratory physiological marker of postoperative cerebrovascular autoregulatory stress after MMD revascularization. In this cohort, the suprasylvian approach was associated with greater POD30 CBF improvement and lower POD30 mRS scores, but also a numerically higher early CHS rate; these findings should be interpreted as early postoperative associations rather than evidence of long-term surgical superiority. The 104.45 cm³ tASL threshold requires internal and external validation before it can be used as a stand-alone clinical decision rule.

## 1 Introduction

Moyamoya disease (MMD) is a chronic, progressive cerebrovascular steno-occlusive disease characterized by the narrowing or occlusion of the terminal portions of the internal carotid arteries and the formation of a fragile collateral vascular network at the base of the brain (Miyamoto et al., 2014; Ahn et al., 2020). Surgical revascularization, primarily through combined direct and indirect bypass techniques, has been established as the standard and most effective treatment. The ultimate goal of such interventions is to safely and effectively restore blood flow to the ischemic distal cortex and deep white matter, thereby arresting the pathological progression of ischemic strokes and mitigating the risk of hemorrhage caused by the rupture of abnormal compensatory neovessels (Fujimura et al., 2022; Gonzalez et al., 2023).

In recent years, with the advancement of microneurosurgical techniques, the strategic selection of anatomical approaches for recipient vessels has garnered widespread attention in the academic community (Shi et al., 2025b). However, the immediate restoration of arterial flow into chronically ischemic vascular beds often disrupts the autoregulatory mechanisms, leading to a dreaded complication known as cerebral hyperperfusion syndrome (CHS) (Tao et al., 2024; Jung et al., 2025). CHS can manifest as severe headaches, seizures, or focal neurological deficits, and in severe cases, it may culminate in devastating intracranial hemorrhage (Gao et al., 2023). Therefore, non-invasive and quantitative evaluation of perioperative cerebral hemodynamics is crucial. Three-dimensional pseudo-continuous arterial spin labeling (3D-pCASL) and territorial arterial spin labeling (tASL) have emerged as powerful, contrast-free magnetic resonance imaging (MRI) modalities capable of precisely mapping cerebral blood flow (CBF) and delineating specific vascular perfusion territories (Wu et al., 2008; Quon et al., 2019; Li et al., 2024).

This study aimed to compare early postoperative clinical outcomes and dynamic hemodynamic evolution between the suprasylvian and infrasylvian surgical approaches using 3D-pCASL and tASL. Because surgical approach was clinically selected rather than randomized, this comparison was designed to describe approach-associated physiological patterns rather than to establish surgical superiority. Furthermore, we sought to evaluate exploratory, physiology-oriented risk signals for postoperative CHS and to clarify how this imaging information may support, but not replace, close clinical monitoring and individualized perioperative management.

## 2 Materials and methods

### 2.1 General data and study design

**Figure 1:**
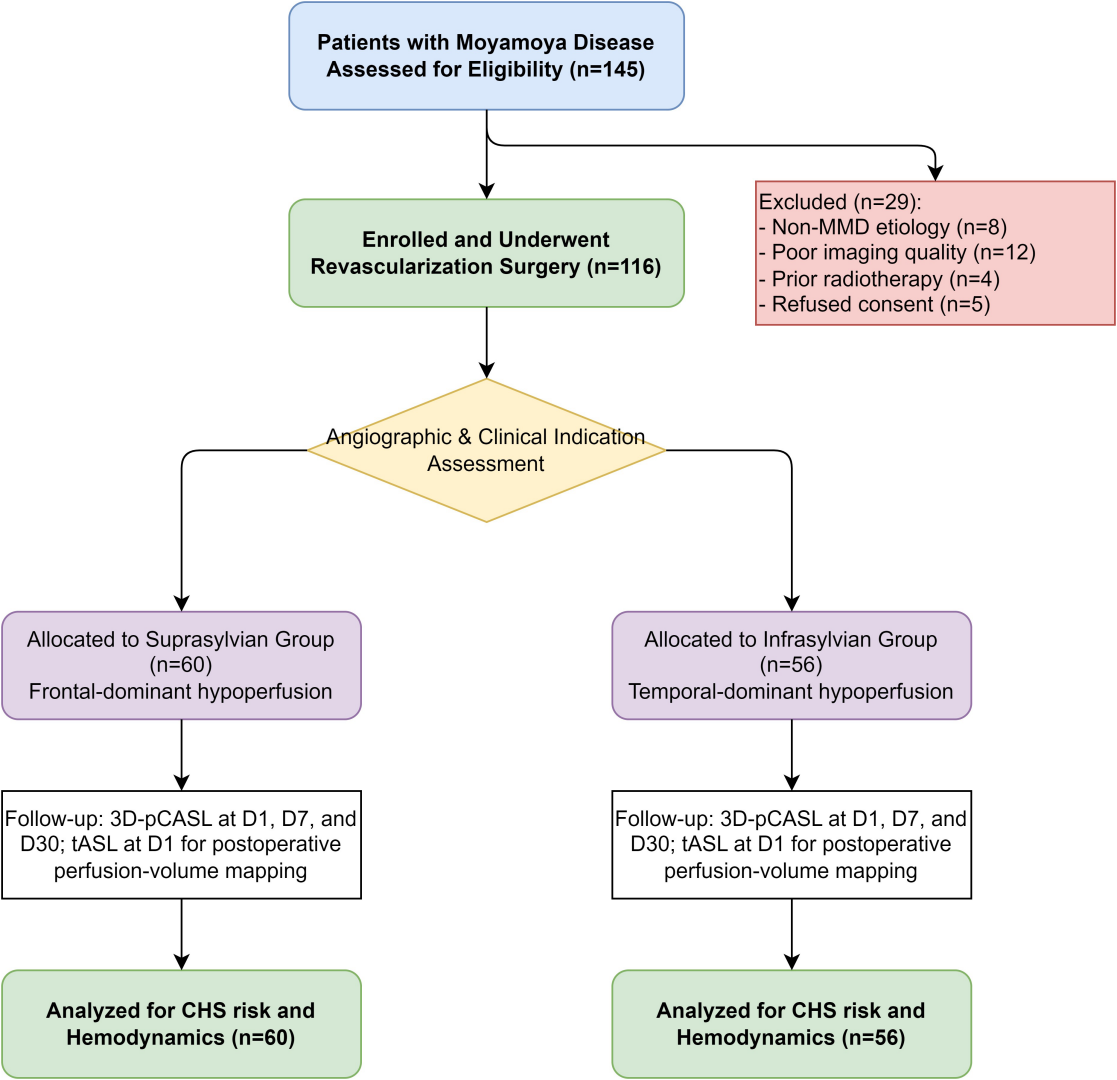
Flowchart of patient enrollment, group allocation, and follow-up in accordance with the Strengthening the Reporting of Observational Studies in Epidemiology (STROBE) guidelines. Patients were allocated to either the suprasylvian or infrasylvian approach based on standardized preoperative angiographic indications and regional hypoperfusion mapping. Follow-up imaging included 3D-pCASL at D1, D7, and D30, with tASL at D1 for postoperative perfusion-volume mapping.

This study selected 116 patients with MMD who underwent combined direct and indirect revascularization at our hospital from June 2023 to March 2025. The study was designed and reported in accordance with the STROBE (Strengthening the Reporting of Observational Studies in Epidemiology) guidelines (von Elm et al., 2007) (see **Figure 1** for the detailed patient enrollment and group allocation flowchart). To standardize clinical decision-making and reduce procedural heterogeneity, the assignment to either the suprasylvian or infrasylvian approach was based on a predefined preoperative angiographic and clinical evaluation of the recipient vessel’s anatomical configuration and hemodynamic compromise (Shi et al., 2025a). Specifically, the suprasylvian approach was preferred when severe hypoperfusion was predominantly located in the frontal lobe and viable M4 recipient branches were identified along the superior margin of the Sylvian fissure. Conversely, the infrasylvian approach was selected for profound temporal lobe ischemia. Based on the surgical approaches, the patients were assigned into two parallel groups: Group 1 (suprasylvian group, n=60) and Group 2 (infrasylvian group, n=56). Because the surgical approach was assigned according to preoperative anatomical and hemodynamic considerations rather than randomization, measured baseline balance cannot exclude residual confounding by indication. Differences in postoperative perfusion or early neurological status were therefore interpreted as approach-associated observational findings rather than causal treatment effects. 

Inclusion criteria: (1) Diagnosed with MMD according to established criteria; (2) Age > 18 years; (3) Successful completion of combined revascularization surgery; (4) Underwent 3D-pCASL imaging before and after surgery; (5) Underwent tASL imaging 1 day postoperatively; (6) Informed consent obtained from patients and their families.

Exclusion criteria: (1) Moyamoya syndrome caused by diseases such as meningitis, Down syndrome, or autoimmune disorders; (2) Inability to tolerate surgical treatment; (3) Inability to cooperate with 3D-pCASL and tASL imaging, or poor image quality; (4) History of cranial radiotherapy. This study was approved by the Ethics Committee of the Second Hospital of Hebei Medical University.

### 2.2 Diagnostic criteria for cerebral hyperperfusion syndrome

According to international consensus and recent radiological criteria, postoperative CHS was strictly defined as the emergence of new-onset severe clinical symptoms (e.g., severe headache, seizures, or transient focal neurological deficits) accompanied by a significant focal increase in CBF at the site of the surgical anastomosis. Specifically, an ipsilateral regional CBF increase of > 100% compared to the preoperative baseline on ASL imaging was required for a definitive radiological diagnosis of CHS (Agarwal et al., 2021; Gao et al., 2023). CHS was assessed in real time during the index hospitalization. When new headache, seizure, altered consciousness, aphasia, motor deficit, or other focal symptoms occurred, the attending neurosurgical team immediately reviewed blood pressure, neurological examination, CT/MRI safety imaging when clinically indicated, and the planned or symptom-triggered ASL findings. Final CHS adjudication was confirmed by consensus between two neurosurgeons and two MRI radiologists who reviewed the perioperative imaging and clinical course. Exact symptom-onset days were retrieved from inpatient neurological records when available. All clinically recognized CHS events occurred before discharge and before the scheduled POD30 assessment; no delayed *de novo* CHS event was identified at POD30.

### 2.3 Methods

#### 2.3.1 3D-pCASL examination

Scanning was performed using a GE SIGNA Architect 3.0T superconducting MRI scanner (GE Healthcare, USA) equipped with a 48-channel phased-array head coil. The scanning sequences included conventional T1-FLAIR, T2WI, T2-FLAIR, diffusion-weighted imaging (DWI), and 3D-pCASL. The 3D-pCASL parameters utilized a fast spin-echo sequence with pseudo-continuous labeling. The labeling region was the cervical carotid artery supply area. The post-labeling delay (PLD) times were set at 1525 ms and 2525 ms. Parameters were: TR/TE = 4795/10.7 ms (PLD = 1525 ms), TR/TE = 5795/10.7 ms (PLD = 2525 ms), with scanning times of 4 min 16 s and 5 min 26 s, respectively.

#### 2.3.2 tASL examination

Using the same GE 3.0T scanner, tASL parameters incorporated a fast spin-echo sequence with pseudo-continuous labeling targeting the cervical carotid supply region. PLD = 1525 ms, TR/TE = 4537/10.7 ms, field of view (FOV) = 240 mm × 240 mm, matrix = 512, slice thickness = 4 mm, number of excitations (NEX) = 2, and a total scan time of 4 min 16 s.

#### 2.3.3 Image analysis

MRI scans were initially analyzed by two experienced MRI radiologists. Post-processing was performed using the ASL software on the AW4.7 workstation provided by the scanner manufacturer, converting grayscale images into CBF pseudo-color maps. Regions of interest (ROIs) were drawn in the middle cerebral artery (MCA) territory, carefully excluding large visible blood vessels, the skull, and the ventricular system. The CBF values of the affected and healthy sides were measured using a mirror-image method. Images at different PLDs were strictly maintained at the same slice levels. The average of three measurements was taken as the final CBF value. For tASL image analysis: post-processing was conducted via the Reformat program on the AW4.7 workstation. Black-and-white images were converted to CBF pseudo-color maps and fused with T2-FLAIR images. The perfusion territory was subsequently delineated to calculate and measure its maximum transverse/longitudinal diameters and total volume. The average of three measurements served as the final volume value.

#### 2.3.4 Definitions and surgical techniques for suprasylvian and infrasylvian approaches

The suprasylvian approach refers to anatomical exposure of the M4 cortical branches (primarily located in the frontal operculum or precentral gyrus region) along the superior margin of the Sylvian fissure via a pterional or frontotemporal craniotomy, performing an end-to-side STA-MCA anastomosis. Surgical steps included: (1) Marking and isolating the frontal or parietal branch of the STA; (2) Creating a 4–6 cm frontotemporal bone flap centered approximately 6 cm above the Sylvian fissure; (3) Opening the dura and performing sharp arachnoid dissection along the superior Sylvian margin under a microscope to expose the largest M4 branch (typically > 0.8 mm); (4) Temporary clipping, fish-mouth incision of the STA, and anastomosis with M4 (10–0 nylon interrupted sutures); (5) Combining with indirect bypass (e.g., EDMS). This approach emphasizes the protection of the superior Sylvian veins and avoidance of excessive frontal lobe retraction.

Conversely, the infrasylvian approach exposes the temporal M4 branches along the inferior margin of the Sylvian fissure. The craniotomy center is shifted slightly temporally, with dissection focused below the superior temporal gyrus. The steps are similar, but great care is taken to protect the vein of Labbé and the temporal cortex.

#### 2.3.5 Perioperative clinical management and outcome adjudication

All patients were managed under a standardized perioperative protocol. Patients were routinely observed in the neurosurgical intensive care or high-dependency unit for at least the first 24–48 h after bypass, followed by inpatient neurological observation; this protocol explains the approximately 2-week hospital stay shown in **Table 1**. Systolic blood pressure was monitored continuously during the immediate postoperative period and then at scheduled ward intervals. No single universal numerical blood-pressure target was applied to all patients; in patients without hyperperfusion symptoms, systolic blood pressure was maintained within an individualized normotensive range according to baseline blood pressure and neurological status. When CHS was suspected or ASL showed marked focal hyperperfusion, the treating team implemented stricter individualized blood-pressure lowering, analgesia/sedation, fluid balance optimization, and repeat neurological assessment. Single antiplatelet therapy with aspirin was continued or initiated after postoperative hemorrhage was excluded and no contraindication was present; clopidogrel or dual antiplatelet therapy was not routinely used. EEG was not performed as a screening test in all patients but was obtained when seizure, impaired consciousness, or non-convulsive seizure activity was clinically suspected. CHS events were recorded relative to the planned D1, D7, and D30 ASL schedule; clinically recognized CHS occurred during the early inpatient postoperative period, and no new CHS event was diagnosed at the D30 assessment.

**Table 1:** Comparison of general data and postoperative hyperperfusion.

Variables	Category	Suprasylvian (n = 60)	Infrasylvian (n = 56)	χ²/t/Fisher	P value
Age (years)	–	46.87 ± 13.65	45.38 ± 11.27	0.639	0.524
Gender	Male	39	37	0.015	0.903
	Female	21	19		
Disease Stage	Stage II	17	19	1.813	0.612
	Stage III	11	14		
	Stage IV	15	11		
	Stage V	17	12		
Smoking History	Yes	14	12	0.060	0.806
	No	46	44		
Hypertension	Yes	14	9	0.961	0.327
	No	46	47		
Diabetes	Yes	8	3	2.147	0.143
	No	52	53		
Preoperative Infarction	Yes	6	3	0.872	0.350
	No	54	53		
Postoperative CHS	Yes	10	3	Fisher exact	0.077
	No	50	53		

#### 2.3.6 Neurological functional scoring

The Modified Rankin Scale (mRS), an internationally recognized tool for assessing post-stroke disability, was utilized. It is graded from 0 to 6: 0 = No symptoms; 1 = No significant disability despite symptoms; 2 = Slight disability, able to look after own affairs without assistance; 3 = Moderate disability, requiring some help; 4 = Moderately severe disability, requiring assistance with bodily needs; 5 = Severe disability, bedridden, incontinent; 6 = Dead. Preoperative mRS was scored within 24 h before surgery, and postoperative mRS was assessed at the scheduled POD30 follow-up used for **Table 1**. Two trained clinicians assigned mRS scores based on structured neurological examination and functional status; disagreements were resolved by consensus. To reduce expectation bias, the final tabulated mRS values were reviewed without access to the tASL perfusion-volume measurements; however, complete blinding to the clinical surgical course was not feasible. The uniform preoperative median mRS of 3.00 (IQR 3.00-3.00) reflects similar baseline global disability in the symptomatic surgical cohort rather than identical neurological deficits in every patient.

### 2.4 Statistical analysis

Data were tabulated in MS Excel and statistically analyzed using SPSS 21.0 and R 4.4.3 packages. Continuous variables were expressed as mean ± standard deviation (SD). Independent-samples t-tests were used for normally distributed continuous variables, and non-parametric tests were used for ordinal neurological scores. Categorical data were expressed as frequencies and analyzed using the Chi-square test or Fisher exact test when event counts were small. To assess the longitudinal evolution of CBF across D0, D1, D7, and D30, two-way repeated-measures ANOVA was used to evaluate time, group, and time-by-group effects; sphericity was assessed with Mauchly’s test when applicable, and Greenhouse–Geisser correction was used when the sphericity assumption was not met. Between-group comparisons at individual time points were performed using independent-samples t-tests as descriptive post hoc comparisons. Because multiple vascular-territory and time-point comparisons were performed, P values from these pointwise comparisons were not adjusted for multiplicity and should be interpreted as nominal exploratory findings. To reduce overfitting from the limited number of CHS events (n=13), Firth penalized logistic regression was used for the exploratory multivariable analysis of CHS risk signals. ROC curves were plotted in the full cohort of 116 patients, including 13 patients with postoperative CHS and 103 without CHS, to determine exploratory cutoff values for critical variables. Spearman rank correlation was applied to explore associations between clinical indices. Statistical significance was set at α = 0.05 (P ≤ 0.05).

## 3 Results

### 3.1 Comparison of baseline characteristics and clinical features

This study included 116 subjects, divided into the “suprasylvian” group (n=60) and the “infrasylvian” group (n=56). Baseline characteristics were summarized and compared to describe measured group balance. The mean age was 46.87 ± 13.65 years in the suprasylvian group and 45.38 ± 11.27 years in the infrasylvian group, with no significant difference (t=0.639, P = 0.524). Gender distribution was also highly consistent (P = 0.903). Disease stages ranged from II to V, showing no statistical difference between groups (P = 0.612). Smoking history, hypertension, diabetes, and preoperative cerebral infarction histories showed no significant inter-group differences (P > 0.05).

Postoperative CHS occurred in 10/60 patients (16.7%) in the suprasylvian group and 3/56 patients (5.4%) in the infrasylvian group. Because only 13 CHS events occurred, Fisher exact test was used for this comparison; the between-group difference did not reach conventional statistical significance (P = 0.077). Therefore, the higher observed CHS rate in the suprasylvian group should be interpreted as a numerical trend rather than definitive evidence that the approach independently increases CHS risk. Residual confounding by indication remains possible because approach selection was not randomized. See **Table 2**. The overall CHS rate was 11.2% (13/116), which is lower than rates reported in some post-bypass neurological deterioration series and should be interpreted alongside the strict clinical-radiological CHS definition, exclusion of poor-quality imaging, and standardized inpatient monitoring. The study by Mukerji et al. addressed transient neurological deficits after STA-MCA bypass and provides useful context for postoperative neurological events, although it did not use the same CHS definition as the present study (Mukerji et al., 2015).

**Table 2:** Comparison of perioperative clinical outcomes and neurological recovery.

Variables	Suprasylvian (n = 60)	Infrasylvian (n = 56)	t/Z	P value
Hospital Stay (Days)	14.57 ± 1.89	13.89 ± 1.88	1.921	0.057
Postoperative Perfusion Volume on POD1 tASL (cm³)	100.50 ± 13.28	97.20 ± 12.77	1.363	0.176
Preoperative mRS	3.00 (3.00, 3.00)	3.00 (3.00, 3.00)	0.161	0.872
Postoperative mRS at POD30	0.00 (0.00, 1.00)	1.00 (0.00, 1.00)	3.627	< 0.001

### 3.2 Comparison of perioperative clinical outcomes and neurological recovery

The mean length of hospital stay was 14.57 ± 1.89 days for the suprasylvian group and 13.89 ± 1.88 days for the infrasylvian group. Although there was an observable numerical difference, it was not statistically significant (t=1.921, P = 0.057). Postoperative perfusion volumes were similar between the suprasylvian (100.50 ± 13.28 cm³) and infrasylvian (97.20 ± 12.77 cm³) groups (P = 0.176).

Non-parametric tests assessed neurological scores. Preoperative mRS medians were comparable between groups, indicating similar baseline global disability rather than identical neurological deficits (Z = 0.161, P = 0.872). At the scheduled POD30 assessment, postoperative mRS was lower in the suprasylvian group than in the infrasylvian group (median 0.00 vs. 1.00; Z = 3.627, P<0.001). Because mRS is a global disability scale and follow-up was limited to POD30, this finding should be interpreted as better early postoperative global functional status rather than long-term neurological recovery or complete reversal of all preoperative neurological or cognitive deficits. See **Table 1**.

### 3.3 Dynamic evolution of CBF in anterior, middle, and posterior cerebral arteries

Using RM-ANOVA, we evaluated CBF changes from D0 to D30 and observed time-dependent postoperative CBF trajectories that differed descriptively between approaches across the measured arterial territories. Because several vascular territories and time points were compared, the pointwise P values are nominal and should be interpreted descriptively. Generally, ACA and PCA flows did not significantly increase on D1 but increased by D7 and D30, which may indicate gradual reduction in vascular resistance and collateral opening.

For the ACA, preoperative and D1 CBF levels were comparable between groups. By D7, the infrasylvian group showed a faster compensatory rise (36.27 ± 4.37 vs. 34.32 ± 3.00 mL/100 g/min, P = 0.006). However, by D30, the trend reversed, with the suprasylvian group reaching a high 43.99 ± 2.91 mL/100 g/min, surpassing the infrasylvian group (P = 0.001).

For the MCA, patient-level showed comparable CBF between the two groups at D0 and D1 (D1: 28.81 ± 3.20 vs. 28.49 ± 2.25 ml/100 g/min, P = 0.539). The suprasylvian group showed a numerically higher MCA CBF at D7 (38.57 ± 2.92 vs. 37.88 ± 2.06 ml/100 g/min, P = 0.146), and the difference became significant by D30 (45.08 ± 2.65 vs. 42.77 ± 1.94 ml/100 g/min, P<0.001).

**Figure 2:**
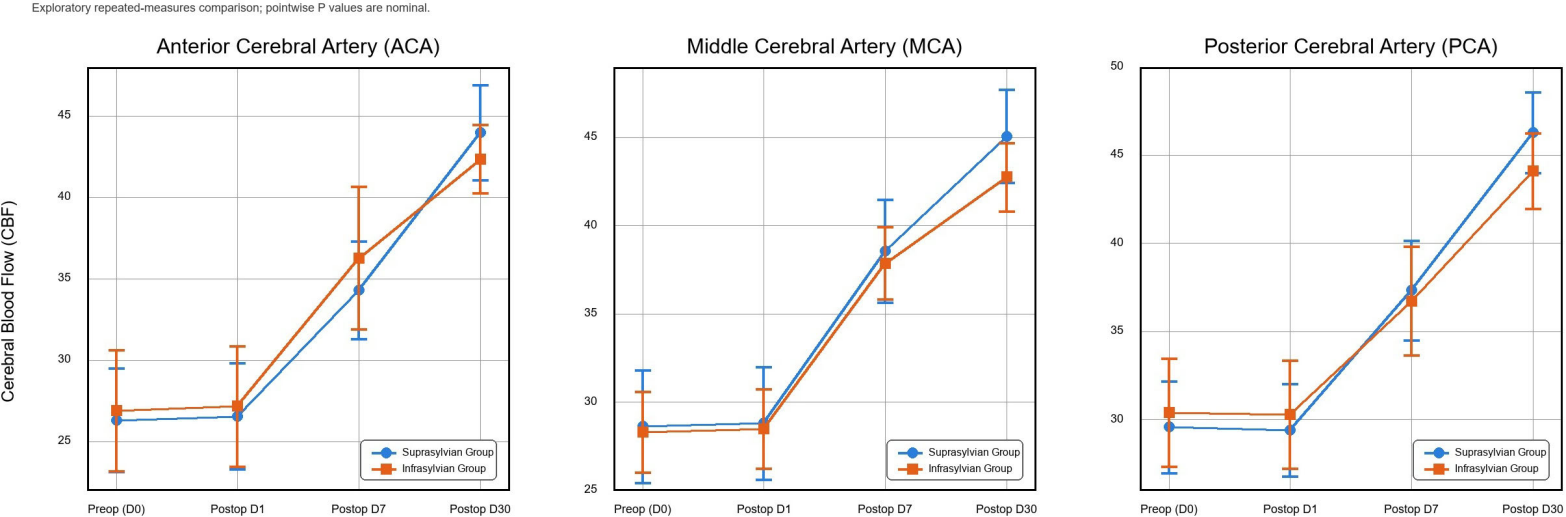
Exploratory dynamic comparison of cerebral blood flow (CBF) in the anterior (ACA), middle (MCA), and posterior (PCA) cerebral arteries measured by 3D-pCASL. CBF was measured preoperatively (D0) and at postoperative days 1, 7, and 30. Error bars indicate standard deviation. Pointwise P values should be interpreted as nominal because multiple territory/time-point comparisons were performed.

PCA perfusion trends showed no significant inter-group differences until D30, when the suprasylvian group showed a higher CBF (46.29 ± 2.29 vs. 44.10 ± 2.14 ml/100 g/min, P<0.001). See **Table 3** and **Figure 2**. **Supplementary Table 1** reports CHS-stratified CBF trajectories and percent-of-D0 values based on the same patient-level dataset used for **Table 3**. The CHS group showed a greater normalized MCA CBF increase at D7 (143.34 ± 9.85% vs. 134.64 ± 16.27%, P = 0.012), while most other regional differences were not statistically significant. Because the CHS subgroup was small (n=13), this analysis was considered exploratory and was not used for confirmatory risk modeling.

**Table 3:** Dynamic evolution of cerebral blood flow and between-group comparisons at each time point.

Artery & Time Point	Suprasylvian (n = 60)	Infrasylvian (n = 56)	t	P value
ACA CBF Preop (D0)	26.32 ± 3.19	26.91 ± 3.72	-0.920	0.359
ACA CBF Postop D1	26.56 ± 3.27	27.17 ± 3.70	-0.952	0.343
ACA CBF Postop D7	34.32 ± 3.00	36.27 ± 4.37	-2.816	0.006
ACA CBF Postop D30	43.99 ± 2.91	42.34 ± 2.10	3.473	0.001
MCA CBF Preop (D0)	28.62 ± 3.18	28.30 ± 2.28	0.614	0.541
MCA CBF Postop D1	28.81 ± 3.20	28.49 ± 2.25	0.616	0.539
MCA CBF Postop D7	38.57 ± 2.92	37.88 ± 2.06	1.465	0.146
MCA CBF Postop D30	45.08 ± 2.65	42.77 ± 1.94	5.315	< 0.001
PCA CBF Preop (D0)	29.59 ± 2.60	30.41 ± 3.06	-1.569	0.119
PCA CBF Postop D1	29.43 ± 2.62	30.31 ± 3.06	-1.662	0.099
PCA CBF Postop D7	37.34 ± 2.82	36.73 ± 3.08	1.105	0.271
PCA CBF Postop D30	46.29 ± 2.29	44.10 ± 2.14	5.326	< 0.001

*Note: Values are presented as mean ± SD. CBF is expressed as mL/100 g/min. Between-group comparisons at each time point were performed using independent-samples t-tests as descriptive post hoc comparisons. Repeated-measures ANOVA was used to assess the longitudinal effects of time, group, and the time-by-group interaction; sphericity was assessed with Mauchly's test when applicable, with Greenhouse-Geisser correction used when required. Pointwise P values are nominal because multiple vascular territories and time points were compared.

### 3.4 Spearman correlation analysis of clinical indicators

**Figure 3:**
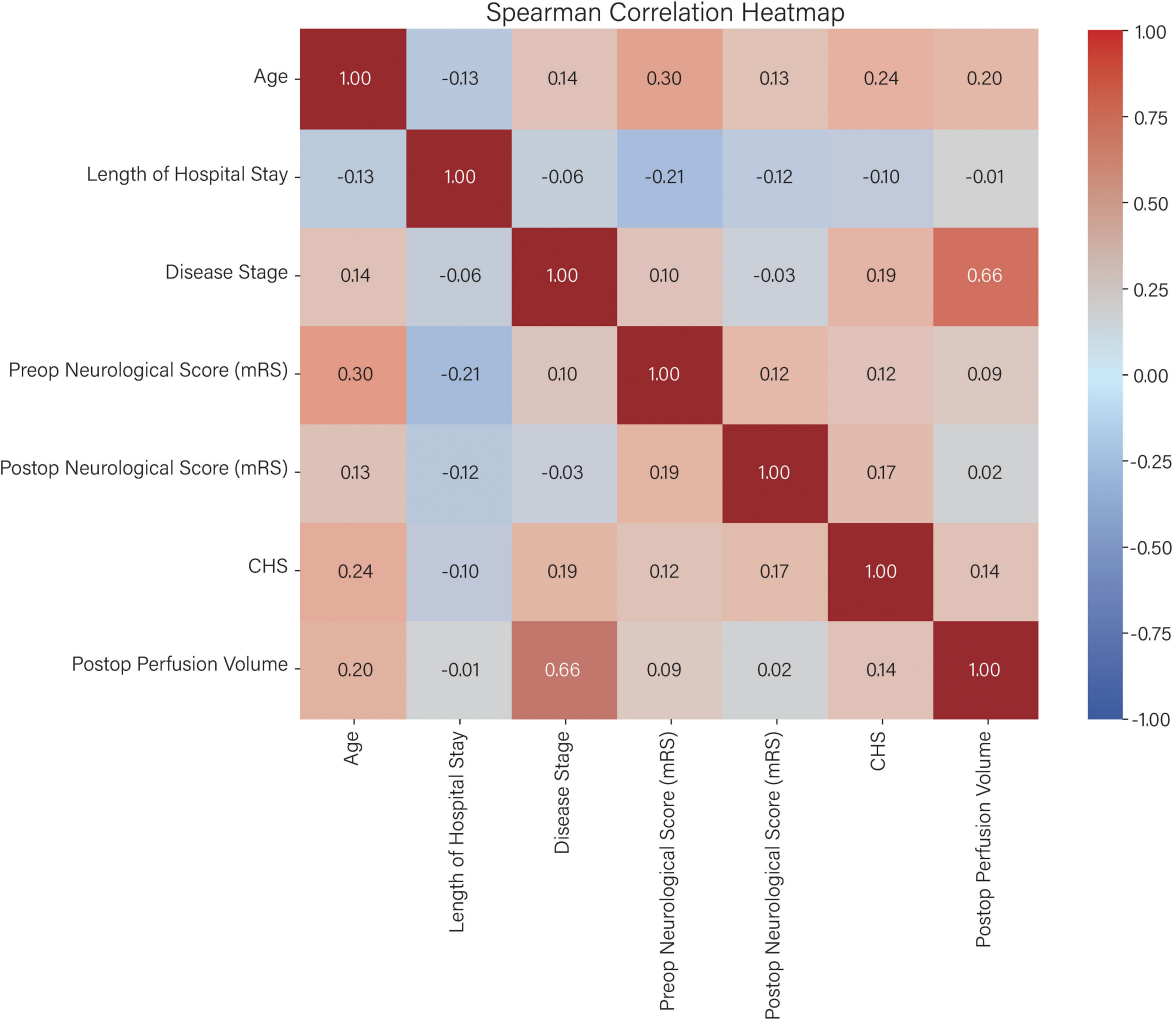
Spearman rank correlation heatmap illustrating the relationships among clinical indicators, patient demographics, preoperative/postoperative neurological scores, disease stages, postoperative perfusion volumes, and the occurrence of cerebral hyperperfusion syndrome (CHS). Positive correlations are depicted in warmer tones, while negative correlations are in cooler tones.

A Spearman rank correlation matrix and heatmap were constructed. A strong positive correlation was identified between disease stage and postoperative perfusion volume (r=0.66), indicating that more advanced angiographic disease was associated with a larger postoperative perfusion territory. Age was weakly positively correlated with preoperative neurological scores (r=0.30) and the incidence of postoperative CHS (r=0.24), which is consistent with, but does not prove, reduced autoregulatory reserve in older patients. CHS also exhibited mild positive correlations with disease stage (r=0.19) and postoperative perfusion volume (r=0.14). See **Figure 3**.

### 3.5 Exploratory Firth penalized logistic regression for CHS risk signals

To reduce model overfitting due to the limited number of CHS events, Firth penalized logistic regression was implemented as an exploratory multivariable model. Because 13 CHS events were modeled against 103 non-CHS cases, estimates and confidence intervals should be interpreted cautiously. The model showed that hypertension was associated with postoperative CHS (penalized OR 17.565, 95% CI 4.371-70.584, P<0.001).

**Figure 4:**
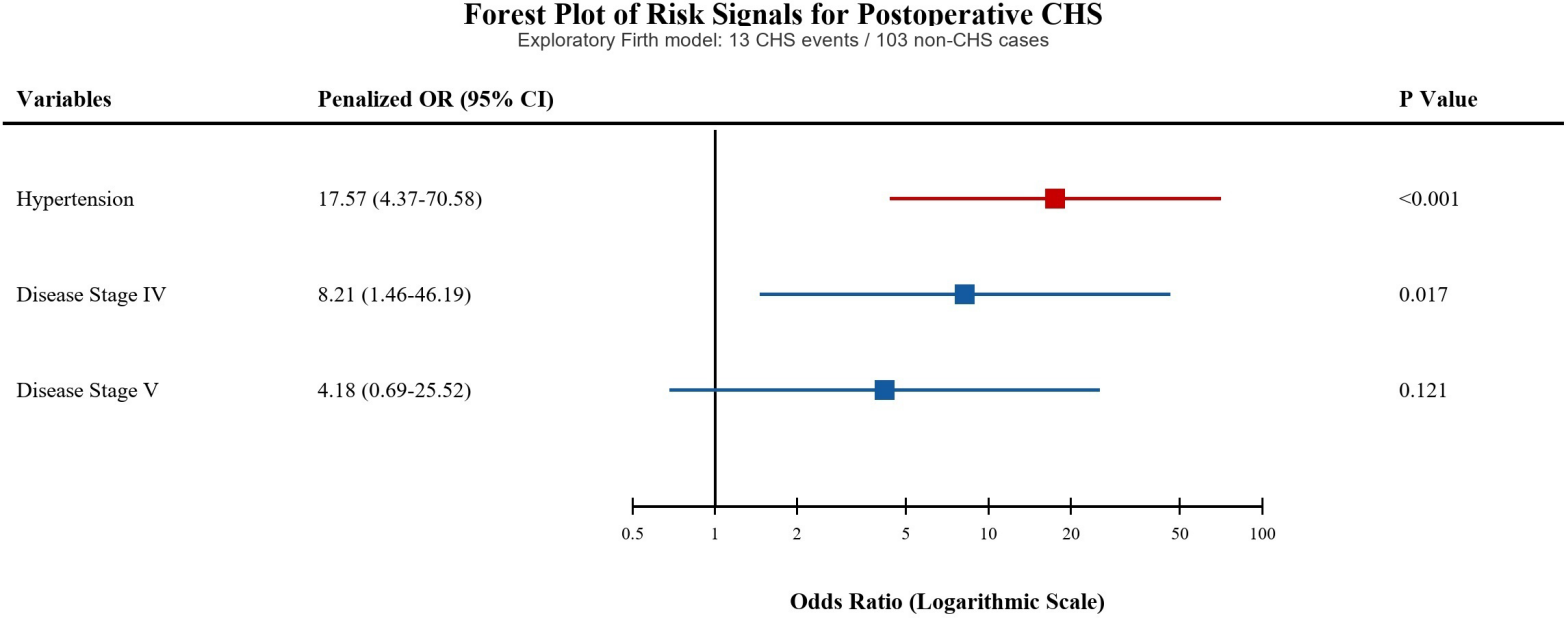
Forest plot summarizing Firth penalized logistic regression risk signals for postoperative cerebral hyperperfusion syndrome (CHS). Odds ratios (OR) and 95% confidence intervals (CI) are shown. The model included 13 CHS events and 103 non-CHS cases; estimates should be interpreted cautiously as exploratory risk signals.

Regarding disease severity, Stage IV showed a positive association with postoperative CHS (penalized OR 8.213, 95% CI 1.460-46.193, P = 0.017), while Stage V showed a positive but statistically imprecise estimate with a confidence interval crossing 1.0 (penalized OR 4.182, 95% CI 0.685-25.524, P = 0.121). Because only 13 CHS events occurred and several confidence intervals were wide, these findings should be interpreted as clinically relevant exploratory risk signals rather than definitive predictors or precise estimates of effect size. See **Table 4** and **Figure 4**.

**Table 4:** Firth penalized logistic regression model for postoperative CHS.

Variables	β coefficient	Penalized OR	95% CI	P value
Hypertension*	**2.866**	**17.565**	**4.371–70.584**	**< 0.001**
Disease Stage IV vs. Stage II/III*	**2.106**	**8.213**	**1.460–46.193**	**0.017**
Disease Stage V vs. Stage II/III	1.431	4.182	0.685–25.524	0.121

*Note: To reduce overfitting due to 13 CHS events and 103 non-CHS cases, Firth's penalized logistic regression was applied. Stage II/III served as the reference category for angiographic stage indicators. Bold values and asterisks indicate P < 0.05. Estimates should be interpreted as exploratory risk signals rather than precise effect-size estimates.

### 3.6 Exploratory ROC analysis for postoperative CHS

Receiver Operating Characteristic (ROC) curves were generated to evaluate the exploratory discrimination of continuous or ordinal variables. Age showed the highest point estimate among the tested ROC variables (AUC 0.748, 95% CI: 0.589-0.908). The exploratory 61-year cutoff provided 69.2% sensitivity and 95.1% specificity, indicating high specificity but limited sensitivity; therefore, age alone cannot be used to exclude CHS.

**Figure 5:**
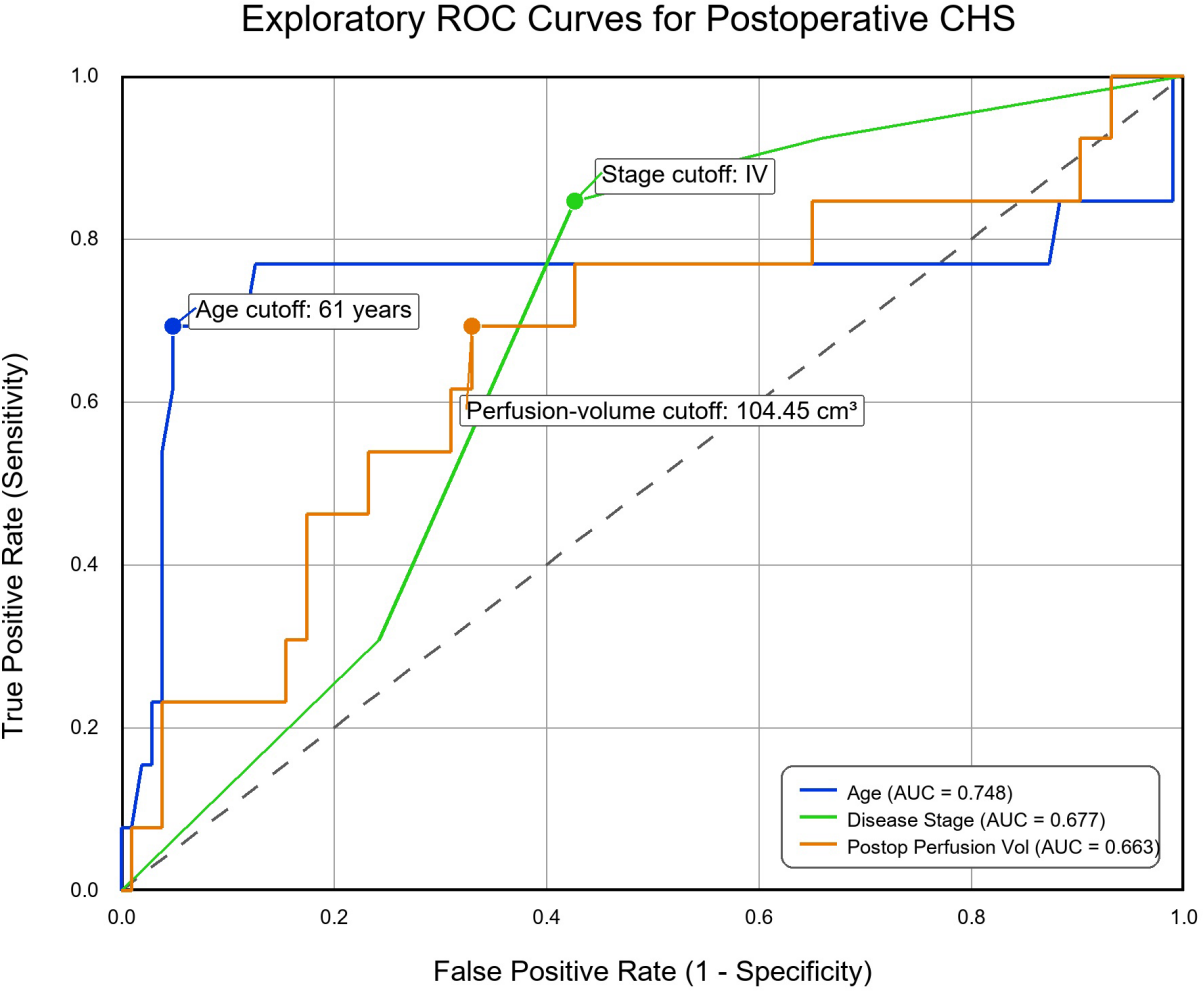
Receiver operating characteristic (ROC) curves for exploratory discrimination of postoperative cerebral hyperperfusion syndrome. AUC values are shown for age, disease stage, and postoperative perfusion volume. Cutoffs were derived and assessed within the same cohort and require validation.

**Figure 6:**
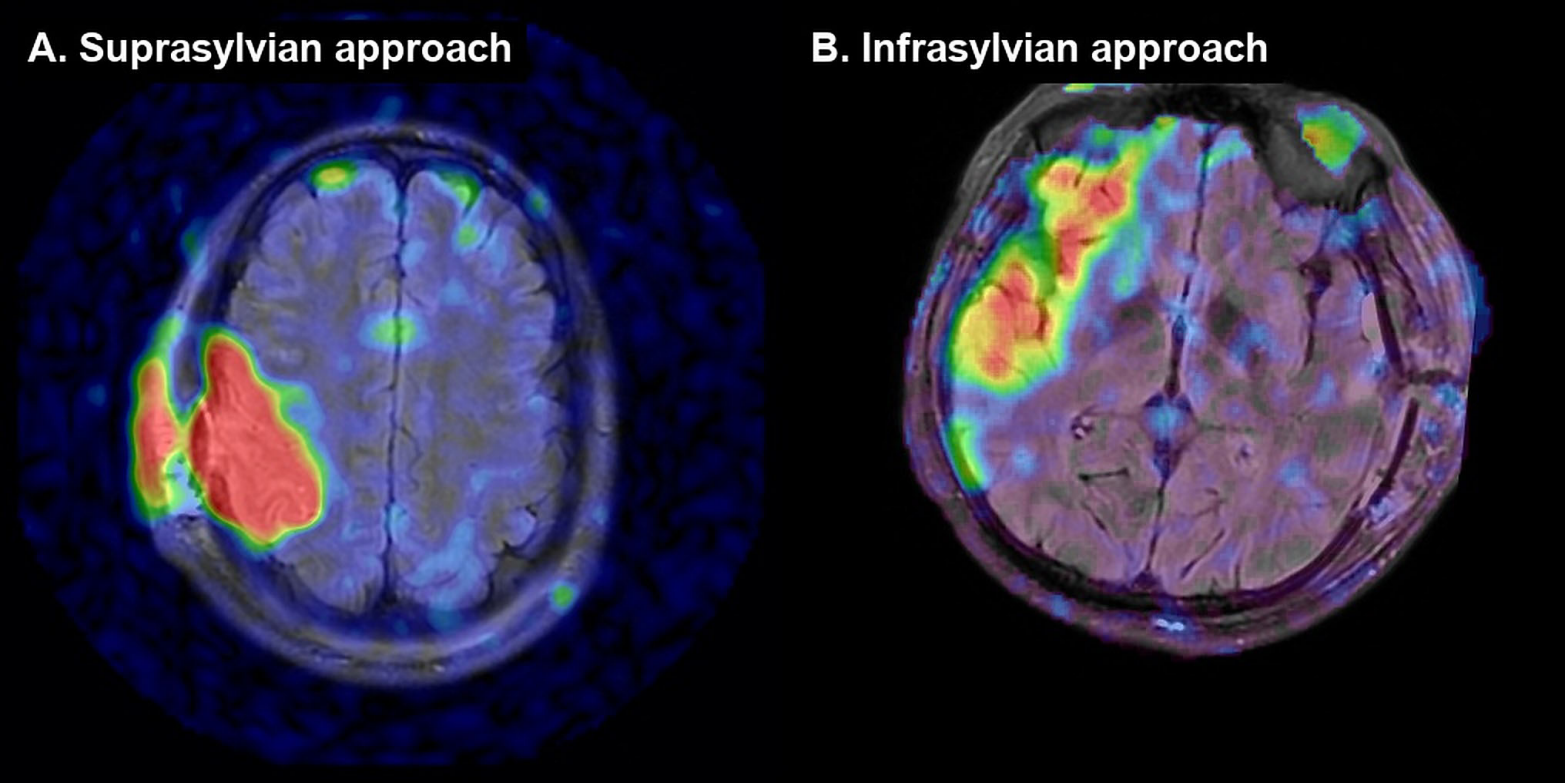
Representative territorial arterial spin labeling (tASL) perfusion maps. **(A)** Postoperative perfusion pattern after the suprasylvian approach. **(B)** Postoperative perfusion pattern after the infrasylvian approach. Warmer color overlays indicate regions of higher postoperative perfusion/focal hyperperfusion.

Disease stage yielded an AUC of 0.677 (95% CI: 0.508-0.845), with an optimal cutoff at Stage IV, offering 84.6% sensitivity and 57.3% specificity. Postoperative perfusion volume showed modest discrimination (AUC 0.663, 95% CI: 0.494-0.832), with a 104.45 cm³ cutoff providing 69.2% sensitivity and 67.0% specificity. These cutoffs were derived and assessed within the same cohort, so their apparent performance may be optimistic. Thus, this tASL cutoff should be interpreted as a hypothesis-generating monitoring trigger rather than a validated decision threshold. See **Table 5**, **Figure 5**, and tASL imaging in **Figure 6**.

**Table 5:** Exploratory ROC analysis for postoperative CHS.

Variables	AUC	95% CI	Youden Index	Cutoff Value	Sensitivity	Specificity
Age	0.748	0.589–0.908	0.644	61 years	69.2%	95.1%
Disease Stage	0.677	0.508–0.845	0.419	Stage IV	84.6%	57.3%
Postop Perfusion Volume	0.663	0.494–0.832	0.362	104.45 cm³	69.2%	67.0%

*Note: ROC analysis was performed using the full cohort of 116 patients, including 13 patients with postoperative CHS and 103 without CHS. Cutoff values were selected by maximizing the Youden index. Because the cutoffs were derived and assessed within the same cohort, they should be considered exploratory and require internal and external validation.

## 4 Discussion

Moyamoya disease is a chronic progressive occlusive cerebrovascular disease that is primarily managed through surgical revascularization when clinically indicated. Its ultimate objective is to achieve safe and effective perfusion of the ischemic distal cortex and deep white matter, thereby arresting the pathological trajectory of ischemic strokes or hemorrhagic events driven by fragile neovascular proliferation (Fujimura et al., 2022; Gonzalez et al., 2023). Recently, the strategic selection of anatomical recipient vessels has garnered attention. In this prospective cohort of 116 MMD patients, the standardized angiographic criteria reduced procedural heterogeneity, but the non-randomized choice of approach means that confounding by indication cannot be fully excluded. By integrating the non-invasive quantitative advantages of 3D-pCASL and tASL technologies (Agarwal et al., 2021; Li et al., 2024), we characterized early postoperative CBF trajectories and evaluated exploratory risk signals for postoperative autoregulatory failure.

Our cohort achieved statistical balance in measured baseline demographics and comorbidities (all P > 0.05). However, baseline balance does not eliminate residual confounding by unmeasured anatomical, collateral, or hemodynamic factors that influenced approach selection. The most clinically direct explanation for the observed CHS pattern is that suprasylvian and infrasylvian bypasses reperfuse cortical territories with different functional eloquence, collateral history, recipient-artery anatomy, and peri-anastomotic hemodynamic exposure. The suprasylvian approach targets MCA frontal/parietal branches, whereas the infrasylvian approach targets the temporal cortex. Our clinical data showed a numerically higher observed CHS rate in the suprasylvian group, although this comparison should be interpreted cautiously because of the small number of CHS events (Fisher exact P = 0.077).

This difference is best interpreted as a difference in peri-anastomotic hemodynamic exposure and clinical detectability rather than proof of intrinsic territory-specific metabolic or microstructural differences. Chronically hypoperfused vascular beds may have impaired vasoreactivity; after direct bypass, abrupt inflow can exceed local autoregulatory reserve and produce symptomatic hyperperfusion in susceptible patients (Gao et al., 2023; Guo et al., 2023). Because this study did not directly measure regional metabolism, microvascular density, or tissue-level vascular reactivity, these mechanisms should be considered plausible explanations rather than demonstrated causal pathways. Residual leptomeningeal collateralization and contralateral hemodynamic stability may also influence clinical expression after unilateral revascularization (Hara et al., 2024; Kang et al., 2024; Kuroha et al., 2024).

The longitudinal CBF data suggested different temporal hemodynamic patterns between the two approaches. Patient-level recalculation showed no significant MCA difference at D1, but the suprasylvian group showed higher ACA, MCA, and PCA CBF by D30. Anatomically, suprasylvian M4 branches may provide a relatively direct route for flow augmentation to the frontoparietal MCA territory. This flow augmentation may contribute to CHS in susceptible patients, but the present observational data do not prove a direct causal pathway. These findings are consistent with improved POD30 perfusion and early postoperative global functional status, as reflected by the lower POD30 mRS in the suprasylvian group, but they do not prove neuronal network reactivation (Sun et al., 2021; Yao et al., 2021; Kronenburg et al., 2023).

CHS can be regarded as a clinically important manifestation of impaired postoperative autoregulation after revascularization. Since the suprasylvian approach may be associated with greater POD30 hemodynamic restoration in selected patients, the clinical dilemma shifts from avoiding a specific approach to identifying and monitoring patients at higher autoregulatory risk. In the Firth penalized model, hypertension was associated with postoperative CHS. Chronic hypertension may impair endothelial function and shift autoregulatory capacity, supporting careful individualized perioperative blood-pressure control.

Additionally, advanced angiographic stage showed a positive risk signal in the penalized model. ROC analysis further extracted exploratory thresholds: age ≥61 years showed high specificity but limited sensitivity, possibly reflecting age-related reduction in vascular compliance and autoregulatory reserve; the present dataset does not allow inference about cerebral amyloid angiopathy. Postoperative tASL perfusion volume ≥104.45 cm³ may reflect a larger territory exposed to abrupt postoperative flow restoration (Zhang et al., 2020; Gao et al., 2023; Yan et al., 2023). Because this threshold was derived and assessed within the same dataset and showed only modest discrimination, it should be regarded as a candidate monitoring signal rather than a clinical decision threshold. If validated in external cohorts, this imaging finding could be used to guide clinical management by prompting closer neurological monitoring, individualized blood-pressure management, and repeat perfusion imaging when symptoms suggest evolving CHS (Rashad et al., 2020; Yokoyama et al., 2023; Endo et al., 2024).

Several limitations should be emphasized. First, approach allocation was not randomized, and differences in postoperative perfusion or POD30 neurological status may partly reflect patient selection, recipient-vessel anatomy, collateral status, or preoperative hemodynamic indications rather than the surgical approach itself. Second, functional outcome was assessed only up to POD30; therefore, the present study cannot support conclusions regarding long-term neurological recovery. Third, only 13 CHS events occurred, so the Firth penalized regression and ROC findings should be regarded as exploratory risk signals with wide uncertainty rather than definitive predictors. Fourth, the 104.45 cm³ perfusion-volume cutoff was derived and assessed within the same cohort and requires internal and external validation. Fifth, formal interobserver or intraobserver reproducibility statistics for tASL perfusion volume, complete exact-day CHS onset distributions, and complete time-resolved postoperative blood-pressure data were not available for all patients. Sixth, multiple vascular-territory and time-point comparisons were performed without formal multiplicity adjustment; nominal P values from these analyses should therefore be interpreted as hypothesis-generating.

## 5 Conclusion

In this prospective cohort, the suprasylvian approach was associated with greater POD30 CBF improvement and lower POD30 mRS scores, but also a numerically higher early CHS rate. These approach-associated findings should be interpreted as early postoperative observational associations, not evidence of long-term superiority or independent causal benefit. Hypertension and advanced angiographic stage were associated with postoperative CHS, while age and tASL-derived postoperative perfusion volume provided exploratory risk-stratification signals. The 104.45 cm³ tASL threshold requires internal and external validation before it can be used as a stand-alone clinical decision rule.

## Data Availability

The original contributions presented in the study are included in the article/**Supplementary Material**. Further inquiries can be directed to the corresponding author.
