# Supplementary material for: Postoperative perfusion volume on territorial arterial spin labeling provides an exploratory risk signal for cerebrovascular autoregulation failure after moyamoya revascularization: a prospective physiological study
Source: Front Physiol. 2026 Jul 29;17:1860201. doi: 10.3389/fphys.2026.1860201 (PMC13461402; doi:10.3389/fphys.2026.1860201)
Supplement: Supplementary file 1 [file Table1.docx]

**Supplementary Table 1: Dynamic Evolution of CBF Stratified by Postoperative CHS Status**

| **Artery and Time Point** | **No CHS (n = 103), CBF ml/100 g/min** | **CHS (n = 13), CBF ml/100 g/min** | **P value** | **No CHS, % of D0** | **CHS, % of D0** | **P value for % of D0** |
| --- | --- | --- | --- | --- | --- | --- |
| ACA CBF D0 | 26.58 ± 3.51 | 26.81 ± 3.11 | 0.811 | 100.00 ± 0.00 | 100.00 ± 0.00 | NA |
| ACA CBF D1 | 26.83 ± 3.54 | 27.09 ± 3.15 | 0.785 | 100.94 ± 2.10 | 101.04 ± 0.99 | 0.785 |
| ACA CBF D7 | 35.36 ± 3.88 | 34.48 ± 3.47 | 0.405 | 135.32 ± 22.99 | 130.39 ± 21.72 | 0.455 |
| ACA CBF D30 | 43.24 ± 2.65 | 42.84 ± 2.95 | 0.646 | 165.61 ± 25.07 | 161.93 ± 23.14 | 0.600 |
| MCA CBF D0 | 28.55 ± 2.82 | 27.80 ± 2.33 | 0.302 | 100.00 ± 0.00 | 100.00 ± 0.00 | NA |
| MCA CBF D1 | 28.74 ± 2.83 | 27.95 ± 2.33 | 0.275 | 100.69 ± 0.92 | 100.54 ± 0.05 | 0.110 |
| MCA CBF D7 | 38.06 ± 2.52 | 39.70 ± 2.47 | 0.039 | 134.64 ± 16.27 | 143.34 ± 9.85 | 0.012 |
| MCA CBF D30 | 43.82 ± 2.59 | 45.08 ± 2.48 | 0.107 | 155.04 ± 18.17 | 163.03 ± 14.27 | 0.083 |
| PCA CBF D0 | 30.07 ± 2.82 | 29.31 ± 3.07 | 0.412 | 100.00 ± 0.00 | 100.00 ± 0.00 | NA |
| PCA CBF D1 | 29.95 ± 2.85 | 29.12 ± 3.01 | 0.359 | 99.59 ± 0.53 | 99.34 ± 0.86 | 0.328 |
| PCA CBF D7 | 37.06 ± 3.01 | 36.95 ± 2.51 | 0.894 | 124.38 ± 16.16 | 127.19 ± 15.13 | 0.540 |
| PCA CBF D30 | 45.24 ± 2.45 | 45.15 ± 2.70 | 0.904 | 151.83 ± 16.96 | 155.21 ± 14.83 | 0.458 |

Note: Values are presented as mean ± SD. The two surgical approaches were combined into a single cohort as requested by the reviewer. Percent-of-D0 values were calculated within each patient as postoperative CBF divided by preoperative CBF × 100. P values were calculated using independent-samples t-tests and should be interpreted as nominal exploratory comparisons because only 13 CHS events occurred and multiple territories/time points were evaluated.
